# Are there sex differences in the variability of fasting metabolism?

**DOI:** 10.1152/japplphysiol.00053.2024

**Published:** 2024-04-18

**Authors:** Louise Bradshaw, Jariya Buniam, James A. Betts, Javier T. Gonzalez

**Affiliations:** ^1^Centre for Nutrition, Exercise and Metabolism, University of Bath, Bath, United Kingdom; ^2^Department for Health, University of Bath, Bath, United Kingdom; ^3^Princess Srisavangavadhana College of Medicine, Chulabhorn Royal Academy, Bangkok, Thailand

**Keywords:** metabolism, sexual dimorphism, variability

## Abstract

There is evidence across species and across many traits that males display greater between-individual variance. In contrast, (premenopausal) females display large within-individual variance in sex hormone concentrations, which can increase within-individual variance in many other parameters. The latter may contribute to the lower representation of females in metabolic research. This study is a pooled secondary analysis of data from seven crossover studies to investigate the between-individual and the within-individual variance in fasting plasma metabolites, resting metabolic rate (RMR), and body mass. Females demonstrated higher within-individual variability of plasma 17β-estradiol [coefficient of variation (CV): 15 ± 15% for males vs. 38 ± 34% for females, *P* < 0.001] and progesterone concentrations (CV: 13 ± 11% for males vs. 52 ± 51% for females, *P* < 0.001) but there were no meaningful differences in the variability of plasma glucose (CV: 4 ± 3% for males vs. 5 ± 5% for females), insulin, lactate, triglycerides (CV: 15 ± 9% for males vs. 15 ± 10% for females), and esterified fatty acid concentrations or in RMR and body mass (CV: 0.43 ± 0.34% for males vs. for 0.42 ± 0.33% females; *P* > 0.05 for all outcomes). Males displayed higher between-individual variance in RMR compared with females (SD: 224 kcal·day^−1^ for males vs. 151 kcal·day^−1^ for females). In conclusion, these data do not provide evidence that females show greater within-individual variability in many fasting metabolic variables, RMR, or body mass compared with males. We conclude that including females in metabolic research is unlikely to introduce greater within-individual variance when using the recruitment and control procedures described in these studies.

**NEW & NOTEWORTHY** To investigate the within-individual variability in metabolic parameters in males and females, we performed a pooled secondary analysis of fasting blood samples, resting metabolic rate, and body mass from seven crossover studies. We found a greater day-to-day variation in 17β-estradiol and progesterone in females compared with males but no meaningful difference in within-individual variability of fasting plasma glucose, insulin, lactate, triglycerides, NEFA, resting metabolic rate, or body mass between females and males.

## Graphical abstract

**Figure:**
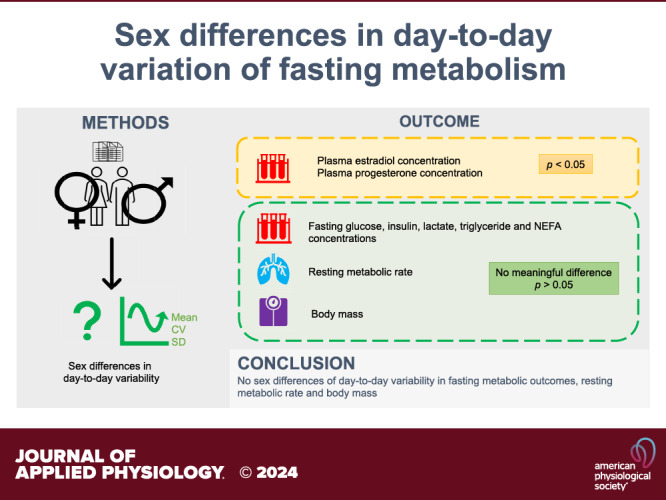

## INTRODUCTION

Understanding the potential sex differences in physiology is important for generalizability of research findings. There are some characteristics for which human females and males are dichotomous and binary; others where there is a relative sex difference in the overall distribution of scores; and others there is no meaningful difference between females and males. In many species, males display greater variation in several morphological and cognitive traits than females. For example, there is greater variation in brain structure in male chimpanzees compared with female chimpanzees (1). In humans, males display greater between-individual variability in energy expenditure compared with females (2). This is despite females having greater within-individual variation in sex hormones, which may have historically contributed to females being underrepresented in human metabolic research (3, 4). The underrepresentation of females in metabolic research could lead to inappropriate recommendations for the female population, resulting in inadequate investigation or treatment of underlying conditions (5–7). For example, before inclusion of females in preclinical drug studies, several drugs were withdrawn from the market due to adverse drug-related health risks in females, which were not apparent during research in males (8). The lack of female representation has led to many funders, such as the National Institutes of Health and scientific journals, now encouraging or requiring both sexes to be included in basic science studies unless given good reason not to (8). Although progress is being made, there is still some evidence for a relative lack of female participants in areas of metabolic research (9).

A particular source of temporal variation (and thus a threat to test-retest reliability) in females is the well-documented monthly rhythm of various outcomes in alignment with the menstrual cycle. For example, females in the luteal phase have been shown to have a lower lipemic response to a high-fat meal (10) and a higher resting metabolic rate (RMR) (11); however, this is not always the case (12). Moreover, if standardizing for menstrual cycle phase or contraceptive use is required, the gold-standard three-step method (13) is costly, increases researcher and participant burden (which may deter females from participating), or may not be possible due to the intervention time scale. In addition, a larger within-individual variance in outcomes in females could result in a larger standard deviation (SD), thus effecting the effect size and reducing statistical power (or increase required sample size). For parallel-group randomized-controlled trials, the within- and between-individual variance are important for power, and for crossover studies, the within-individual (within-individual) variance is important. Reasons to select a sample that will give a smaller SD will mean that fewer resources are needed to answer a research question and can therefore be considered more justifiable ethically. However, if populations are excluded on a false assumption of greater variability (between-individual and/or within-individual), then this may be reducing generalizability of the study unnecessarily.

Although it is possible that females exhibit greater variance than males across multiple days of repeated metabolic testing, this remains to be empirically tested. Specifically, to the authors’ knowledge, no previous study has compared daily variance in systemic metabolites, resting metabolic rate, or body mass (BM) between human females and males. If that within-individual variance is not different between sexes, then excluding females on the basis of greater heterogeneity in response to the challenges of standardization or the potential reduction of statistical power would clearly not be valid arguments. The aim of this study was therefore to determine whether sex differences exist in relation to the within-individual variance in fasted systemic metabolites and other common outcomes for metabolic studies, such as resting metabolic rate and body mass. A secondary aim was to assess whether between-individual variance was greater in males versus females (in line with the greater male variability hypothesis). We hypothesize that males will display greater between-individual variability and females will display greater within-individual variability.

## METHODS

### Study Design

**Figure 1.**
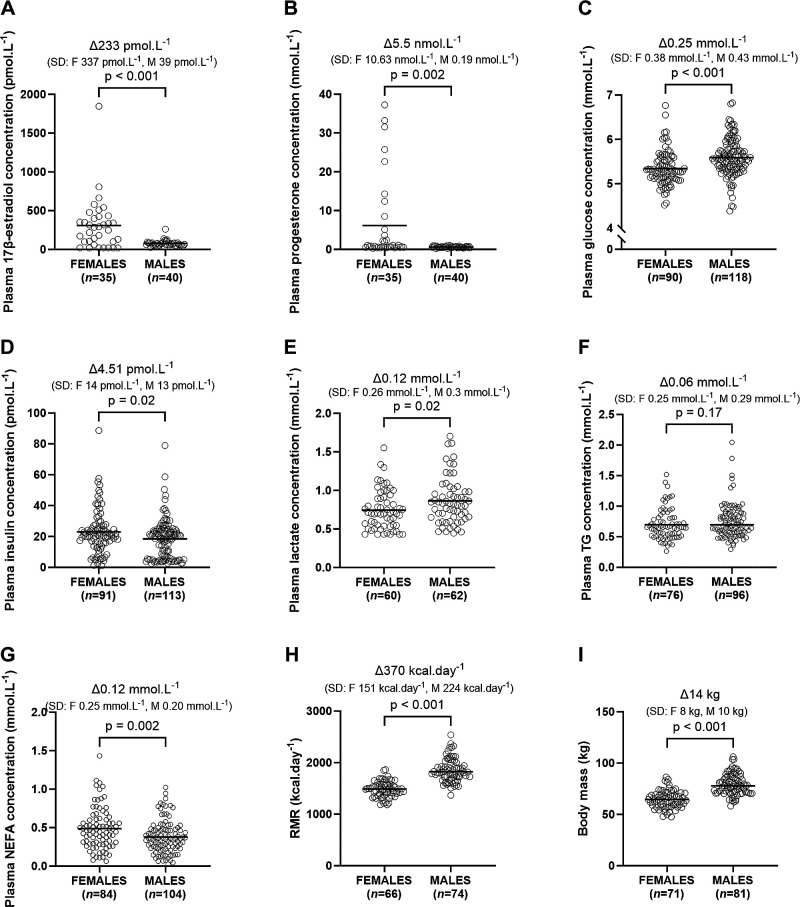
Mean concentrations for fasting plasma 17β-estradiol (*A*), progesterone (*B*), glucose (*C*), insulin (*D*), lactate (*E*), triacylglycerol (TG; *F*), nonesterified fatty acids (NEFA; *G*), resting metabolic rate (RMR; *H*), and body mass (*I*) in males and females. Data were analyzed by *t* test and are shown as means.

**Figure 2.**
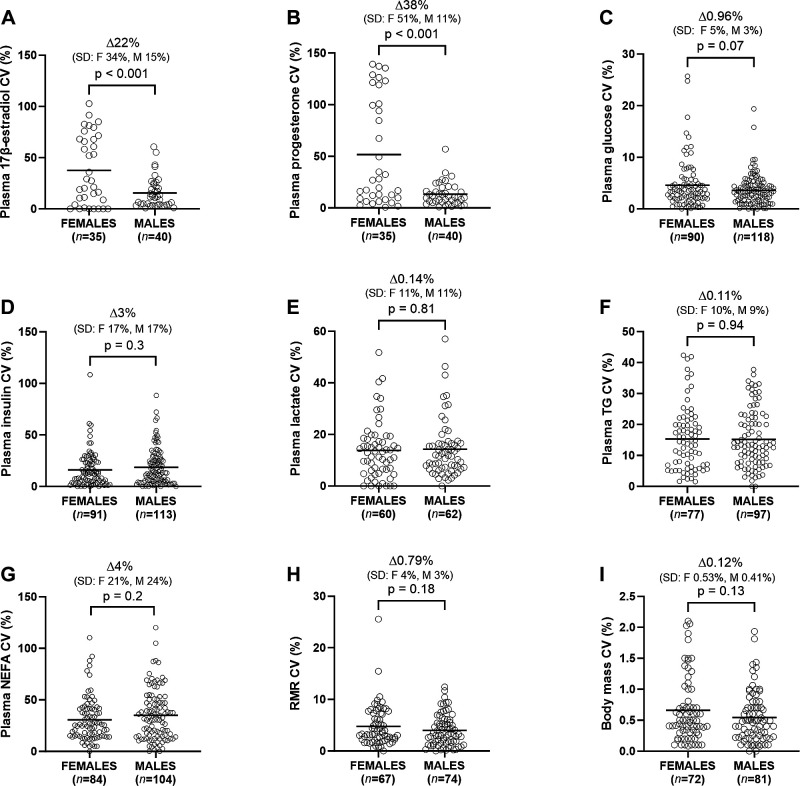
Within-individual coefficient of variation (CV) for fasting plasma 17β-estradiol (*A*), progesterone (*B*), glucose (*C*), insulin (*D*), lactate (*E*), triacylglycerol (TG; *F*), nonesterified fatty acids (NEFAs; *G*), resting metabolic rate (RMR; *H*), and body mass (*I*) in males and females. Data were analyzed by *t* test and are shown as means.

**Figure 3.**
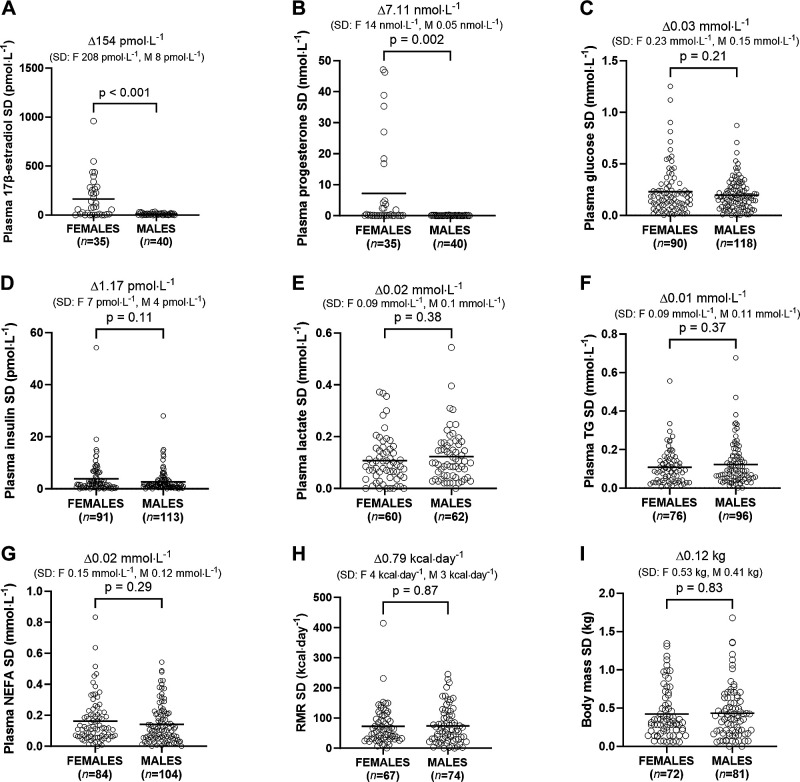
Within-individual standard deviation (SD) for fasting plasma 17β-estradiol (*A*), progesterone (*B*), glucose (*C*), insulin (*D*), lactate (*E*), triacylglycerol (TG; *F*), nonesterified fatty acids (NEFAs; *G*), resting metabolic rate (RMR; *H*), and body mass (*I*) in males and females. Data were analyzed by *t* test and are shown as means.

This study is a pooled secondary analysis from 7 independent studies that investigated metabolic outcomes in >200 females and males (14–18; J. J. D. Watkins, H. A. Smith, A. Hengist, S. B. Nielse, U. R. Mikkelsen, J. Saunders, F. Koumanov, J.A. Betts, J.T. Gonzalez, unpublished observations; S. Carter, B. Spellanzon, L. Bradshaw, E. Johnson, F. Koumanov, A. Moreno-Cabañas, J.A. Betts, D. Thompson, L. Hodson, J. T. Gonzalez, unpublished observations). All studies were registered at ClinicalTrials.gov (NCT03232034, NCT03029364, NCT03509610, NCT03819972, NCT04659902, NCT04924530, and NCT03324191). The studies included test-retest measures from 2 to 4 separate days of testing. All studies included some level of standardization of diet and abstinence from vigorous activity for at least 24 h before testing. Full details of pretrial and menstrual cycle standardization are shown in Table 1. Data from fasted baseline blood samples, taken between 07:00 and 09:00 am, including plasma 17β-estradiol, progesterone, insulin, glucose, triglycerides (TGs), nonesterified fatty acids (NEFAs), and lactate concentrations, along with whole body measures of resting metabolic rate (RMR) and body mass, were included in this analysis. Plasma samples from all studies were analyzed using the same assays. Plasma glucose, TG, NEFA, and lactate concentrations were measured using an automated analyzer (Daytona Rx, Randox Laboratories, UK). Plasma insulin concentrations were measured using enzyme-linked immunosorbent assay kits (Mercodia AB, Sweden). 17β-Estradiol and progesterone concentrations were measured using electrochemiluminescence immunoassay on aRoche Cobas e602 (Roche Diagnostics, Switzerland). The number of participants in each analysis shown is reported in Figs. 1, 2, and 3, but ranged from 35 to 91 for females and 40 to 118 for males. Both the female and male participants in this analysis had a mean age of 30 and median age of 26.

**Table 1.** Study characteristics and within-individual coefficient of variation and standard deviation for fasting metabolic outcomes, resting metabolic rate, and body mass for individual studies

Study	Sample Size	Age, yr	Pretrial Standardization	Number of Trials	Washout Period	Menstrual/Contraceptive Control	Coefficient of Variation, %		Standard Deviation	
S. Carter, B. Spellanzon, L. Bradshaw, E. Johnson, F. Koumanov, A. Moreno-Cabañas, J.A. Betts, D. Thompson, L. Hodson, J. T. Gonzalez, unpublished observations	*n* = 26 M = 12 F = 14	24 ± 6 (18–43)	Participants recorded diet and physical activity for 48 h before the first trial and replicated these for the 48 h before subsequent trials. Abstained from alcohol, caffeine, and vigorous activity for 24 h prior to each trial	3	28 ± 7 days	Premenopausal, hormonal contraceptive users included. No control for phase of menstrual cycle	Glucose Insulin Lactate TG NEFA RMR	M 3.29 (1.58) F 4.50 (3.46) *P* = 0.28 M 32.17 (19.23) F 31.47 (29.96) *P* = 0.95 M 13.98 (7.51) F 17.61 (11.22) *P* = 0.37 M 16.42 (9.11) F 19.31 (9.75) *P* = 0.47 M 20.54 (13.57) F 30.59 (26.3) *P* = 0.26 M 5.11 (1.75) F 6.98 (4.09) *P* = 0.17	Glucose Insulin Lactate TG NEFA RMR	M 0.15 (0.10) F 0.15 (0.20) *P* > 0.99 M 5.38 (4.7) F 9.5 (14.46) *P* = 0.36 M 0.13 (0.08) F 0.14 (0.10) *P* = 0.82 M 0.12 (0.09) F 0.11 (0.06) *P* = 0.69 M 0.06 (0.04) F 0.13 (0.14) *P* = 0.11 M 101.5 (44.85) F 136.2 (105.6) *P* = 0.33
Chen et al. (14)	*n* = 20 M = 14 F = 6	26 ± 7 (20–50)	Participants recorded diet and physical activity for 24 h prior to the first trial and replicated this for 24 h prior to all trials. Refrained from tea, coffee, alcohol consumption, and vigorous physical activity for 24 h before each trial	3	Between 48 h and 7 days for males and females using oral contraception, and tested during the follicular phase of the menstrual cycle for menstruating females	Oral contraception or visit in the follicular phase of the menstrual cycle (3–12 days after the first menses)	Glucose RMR BM	M 2.97 (1.68) F 4.24 (2.74) *P* = 0.22 M 3.28 (2.44) F 4.39 (3.01) *P* = 0.39 M 0.53 (0.31) F 0.34 (0.37) *P* = 0.26	Glucose RMR BM	M 0.16 (0.09) F 0.21 (0.13) *P* = 0.30 M 68.0 (44.44) F 81.35 (44.64) *P* = 0.55 M 0.41 (0.23) F 0.22 (0.25) *P* = 0.12
Chrzanowski-Smith et al. (18)	*n* = 73 M = 41 F = 32	37 ± 11 (19–63)	Overnight fast (12 ± 1 h); participants abstained from alcohol and strenuous physical activity for 48 h, and replicated diet and physical activity for 48 h before the second trial	2	Between 7 and 28 days	Trials at same phase of menstrual cycle in eumenorrheic females	Glucose Insulin Lactate TG NEFA RMR BM Estrogen Progesterone	M 3.45 (3.45) F 4.9 (5.48) *P* = 0.17 M 5.42 (5.21) F 5.6 (4.5) *P* = 0.87 M 14.9 (13.16) F 13.15 (12.57) *P* = 0.56 M 12.38 (9.35) F 13.01 (10.23) *P* = 0.78 M 29.60 (21.33) F 31.05 (21.2) *P* = 0.77 M 4.15 (3.53) F 3.78 (2.81) *P* = 0.62 M 0.51 (0.36) F 0.63 (0.55) *P* = 0.28 M 15.55 (14.74) F 34.62 (33.65) *P* < 0.001 M 13.29 (11.10) F 51.69 (51.03) *P* < 0.001	Glucose Insulin Lactate TG NEFA RMR BM Estrogen Progesterone	M 0.19 (0.18) F 0.26 (0.28) *P* = 0.25 M 1.28 (1.54) F 1.21 (1.06) *P* = 0.81 M 0.12 (0.11) F 0.09 (0.09) *P* = 0.21 M 0.08 (0.06) F 0.08 (0.06) *P* = 0.85 M 0.1 (0.11) F 0.14 (0.14) *P* = 0.16 M 75.86 (67.8) F 54.77 (41.91) *P* = 0.12 M 0.4 (0.29) F 0.4 (0.35) *P* = 0.95 M 9.97 (8.17) F 164.2 (207.8) *P* < 0.001 M 0.07 (0.05) F 7.18 (14.07) *P* = 0.002
Hengist et al. (15)	*n* = 25 M = 10 F = 15	26 ± 8 (18–48)	Refrain from vigorous physical activity in the 24 h before each visit. Participants provided with food from menu and recorded actual intake for 24 h and replicated before each trial	3	13 (7–21) days for males and females using oral contraception, and 28 (28–34) days for menstruating females	Oral contraception or visit at the same phase of cycle	Glucose Insulin Lactate TG NEFA RMR BM	M 3.34 (1.48) F 3.55 (1.69) *P* = 0.76 M 14.20 (12.09) F 19.54 (14.87) *P* = 0.36 M 12.05 (4.73) F 12.29 (6.45) *P* = 0.92 M 20.31 (9.80) F 17.37 (10.82) *P* = 0.5 M 24.46 (13.87) F 21.17 (11.68) *P* = 0.54 M 2.92 (2.12) F 4.35 (2.61) *P* = 0.16 M 0.69 (0.66) F 0.92 (0.6) *P* = 0.38	Glucose Insulin Lactate TG NEFA RMR BM	M 0.18 (0.09) F 0.19 (0.09) *P* = 0.9 M 3.83 (4.01 F 7.03 (6.01) *P* = 0.16 M 0.14 (0.05) F 0.14 (0.10) *P* = 0.87 M 0.19 (0.12) F 0.15 (0.14) *P* = 0.49 M 0.09 (0.06) F 0.10 (0.06) *P* = 0.62 M 45.91 (40.73) F 63.62 (35.85) *P* = 0.26 M 0.54 (0.55) F 0.56 (0.35) *P* = 0.89
Perkin et al. (17)	*n* = 50 M = 34 F = 16	26 ± 7 (18–50)	Participants recorded physical activity and diet for 48 h and replicated for 48 h ahead of subsequent visits	4	Minimum of 2 days between trials	Tested within a week of the same day of their menstrual cycle on all occasions	Glucose Insulin TG NEFA	M 3.94 (2.89) F 2.88 (1.46) *P* = 0.22 M 32.77 (17.15) F 26.42 (14.33) *P* = 0.21 M 16.58 (8.18) F 15.79 (9.42) *P* = 0.76 M 51.72 (26.04) F 38.26 (26.45) *P* = 0.1	Glucose Insulin TG NEFA	M 0.21 (0.15) F 0.15 (0.07) *P* = 0.16 M 1.6 (1.11) F 1.48 (0.86) *P* = 0.71 M 0.15 (0.14) F 0.13 (0.09) *P* = 0.64 M 0.24 (0.12) F 0.29 (0.18) *P* = 0.31
Watkins et al. (16)	*n* = 18 M = 8 F = 10	34 ± 18 (18–64)	Participant recorded diet for 24 h prior to the first trial and replicated this for 24 h prior to each subsequent trial. Refrained from caffeine, alcohol, and vigorous physical activity for 24 h prior to each trial	4	Between 2 and 7 days	None	Glucose Insulin NEFA BM	M 5.14 (2.19) F 8.77 (8.33) *P* = 0.25 M 17.83 (11.02) F 14.56 (6.16) *P* = 0.48 M 26.92 (19.57) F 30.99 (12.67) *P* = 0.63 M 0.74 (0.50) F 0.63 (0.43) *P* = 0.63	Glucose Insulin NEFA BM	M 0.28 (0.13) F 0.45 (0.38) *P* = 0.24 M 8.74 (8.78) F 6.07 (4.19) *P* = 0.45 M 0.09 (0.07) F 0.14 (0.07) *P* = 0.18 M 0.66 (0.45) F 0.46 (0.29) *P* = 0.27
J. J. D. Watkins, H. A. Smith, A. Hengist, S. B. Nielse, U. R. Mikkelsen, J. Saunders, F. Koumanov, J.A. Betts, J. T. Gonzalez, unpublished observations	*n* = 15 M = 9 F = 6	24 ± 5 (18–34)	Participants recorded diet for 24 h prior to first trial and replicated this diet for 24 h prior to each subsequent trial. Refrained from caffeine, alcohol, and any vigorous physical activity for 24 h prior to each trial	4	Between 2 and 7 days	None	Insulin BM	M 11.18 (8.25) F 12.49 (8.97) *P* = 0.78 M 0.37 (0.23) F 0.56 (0.20) *P* = 0.13	Insulin BM	M 2.81 (2.25) F 4.03 (3.21) *P* = 0.40 M 0.29 (0.19) F 0.34 (0.12) *P* = 0.56

Data were analyzed by *t* test and are shown as means (SD). Washout period refers to time between trial days. BM, body mass; NEFA, nonesterified fatty acid; RMR, resting metabolic rate.

### Statistical Analysis

Mean values from multiple trial days were calculated for each participant, and overall mean or median was calculated for females and males of each outcome. The within-individual coefficient of variation (CV) and standard deviation (SD) were calculated for each participant. The overall mean CV and SD of each outcome were calculated for females and males. As outliers in the data are a legitimate element of potential sex differences, we opted to use only means in the analysis, and the normality of the data was not assessed. HOMA-IR was calculated from plasma glucose and insulin concentrations using the following equation: [plasma insulin concentration (µU/L) × plasma glucose concentration (mmol/L)]/22.5 (19). To assess differences between sexes, independent *t* tests were performed on the full dataset for the mean, SD, and CV of each outcome. To identify the effects of different standardization protocols on sex differences, independent *t* tests were also performed on individual outcomes from each individual trial. Statistical significance was defined as *P* ≤ 0.05. Biologically meaningful differences were defined as 0.5 mmol/L for glucose (20), 6 pmol/L for insulin (21), 0.29 mmol/L for NEFA (22), 0.5 mmol/L for lactate (15), and 0.09 mmol/L for TG (23).

## RESULTS

### Sex Differences in Metabolites, RMR, and Body Mass

Females demonstrated a higher mean concentration of plasma 17β-estradiol (*P* < 0.001; Fig. 1*A*), plasma progesterone (*P* = 0.002; Fig. 1*B*), plasma insulin (*P* = 0.02; Fig. 1*D*), and plasma NEFA (*P* = 0.002; Fig. 1*G*) compared with males. Conversely, females demonstrated a lower mean concentration of plasma glucose (*P* < 0.001; Fig. 1*C*) and plasma lactate (*P* = 0.02; Fig. 1*E*) and a lower mean of resting metabolic rate (*P* < 0.001; Fig. 1*H*) and body mass (*P* < 0.001; Fig. 1*I*). There was no significant or biologically meaningful difference between females and males of mean plasma TG concentration (*P* = 0.17; Fig. 1*F*) or HOMA-IR (*P* = 0.08; Supplemental Fig. S1*A*).

### Sex Differences in within-Individual Variability

Females displayed a higher within-individual CV in plasma 17β-estradiol (*P* < 0.001; Fig. 2*A*) and plasma progesterone (*P* < 0.001; Fig. 2*B*) compared with males. Differences in the within-individual CV of plasma glucose (*P* = 0.07; Fig. 2*C*), plasma insulin (*P* = 0.3; Fig. 2*D*), plasma lactate (*P* = 0.81; Fig. 2*E*), plasma TG (*P* = 0.94; Fig. 2*F*), plasma NEFA (*P* = 0.2; Fig. 2*G*), and HOMA-IR (*P* = 0.24; Supplemental Fig. S1*C*) between males and females were not significant or biologically meaningful (difference in means all <1.5%). Similarly, differences in the within-individual CV of resting metabolic rate (*P* = 0.18; Fig. 2*H*) and body mass (*P* = 0.13; Fig. 2*I*) between males and females were also not significant or meaningful (difference in medians both <0.1%).

Females displayed a higher within-individual SD for plasma 17β-estradiol (*P* < 0.001; Fig. 3*A*) and progesterone (*P* = 0.002; Fig. 3*B*) compared with males. Differences in the within-individual SD of plasma glucose (*P* = 0.21; Fig. 3*C*), plasma insulin (*P* = 0.11; Fig. 3*D*), plasma lactate (*P* = 0.38; Fig. 3*E*), plasma TG (*P* = 0.37; Fig. 3*F*), plasma NEFA (*P* = 0.29; Fig. 3*G*), and HOMA-IR (*P* = 0.19; Supplemental Fig. S1*B*) between females and males were not significant or biologically meaningful. Similarly, differences between females and males in the within-individual SD for resting metabolic rate (*P* = 0.87; Fig. 3*H*) and body mass SD (*P* = 0.83; Fig. 3*I*) were also not significant or biologically meaningful (difference in means <10 kcal/day and 0.1 kg, respectively).

### Sex Differences in Between-Individual Variability

Females demonstrated higher between-individual variance in plasma 17β-estradiol (SD: 337 pmol·L^−1^ for females vs. 39 pmol·L^−1^ for males; Fig. 1*A*) and plasma progesterone concentrations (SD: 10.63 nmol·L^−1^ for females vs. 0.19 nmol·L^−1^ for males; Fig. 1*B*) compared with males. There was no difference in between-individual variance in plasma glucose (SD: 0.38 mmol·L^−1^ for females vs. 0.43 mmol·L^−1^ for males; Fig. 1*C*), plasma insulin (SD: 14 pmol·L^−1^ for females vs. 13 pmol·L^−1^ for males; Fig. 1*D*), plasma lactate (SD: 0.26 mmol·L^−1^ for females vs. 0.3 mmol·L^−1^ for males; Fig. 1*E*), plasma TG (SD: 0.25 mmol·L^−1^ for females vs. 0.29 mmol·L^−1^ for males; Fig. 1*F*), plasma NEFA concentrations (SD: 0.25 mmol·L^−1^ for females vs. 0.2 mmol·L^−1^ for males; Fig. 1*G*), and HOMA-IR (SD: 0.51 for females vs. 0.46 for males; Supplemental Fig. S1*A*) between females and males. Males displayed higher between-individual variance in RMR compared with females (SD: 224 kcal·day^−1^ for males vs. 151 kcal·day^−1^ for females; Fig. 1*H*) but there was no difference in between-individual variance in BM between sexes (SD: 8 kg for females vs. 10 kg for males; Fig. 1*I*).

The overall data were consistent with the observations within each study, as demonstrated by no evidence of meaningful differences between males and females in the within-individual CV or the SD of individual outcomes in each study apart from plasma 17β-estradiol and progesterone concentrations (Table 1).

## DISCUSSION

These data do not provide evidence that, when instructed to standardize lifestyle factors (e.g., diet and physical activity) in the days prior to testing and when menstrual cycle phase is controlled (repeated trials 28 days apart), there are sex differences in the within-individual variation in various fasting metabolic variables, including plasma glucose, insulin, lactate, triacylglycerol, and esterified fatty acid concentrations, or in the resting metabolic rate or body mass. This was despite observing the expected greater variation in sex hormone concentrations in females compared with males. Within the context of the studies presented (i.e., inclusion/exclusion criteria and pretrial standardization), these data do not support the exclusion of females from metabolic research on the basis of introducing greater within-individual variance in the fasting outcomes described in this report. There was a trend for higher CV of plasma glucose concentrations in females compared with males. Estrogen plays a crucial role in regulating fat and glucose metabolism in females, which may result in potential variations of metabolic parameters across the menstrual cycle (24–26). Previous literature has reported higher blood glucose concentrations in the luteal phase compared with the follicular phase (27). A higher within-individual variability of plasma glucose in females may be observed if there was no standardization of menstrual cycle and females were tested in different phases. By promoting inclusivity without introducing greater variance, more generalizable inferences can be obtained without a disproportionate increase in resource requirements. Whether females display higher (or lower) within-individual variation in the underlying fluxes or in postprandial or exercise responses remains to be assessed. Moreover, it remains unclear whether testing specifically within different phases of the menstrual cycle would result in greater within-individual variability.

These data show that females display higher fasting concentrations of insulin and NEFA but a lower fasting glucose concentration, resting metabolic rate, and body mass, which are all well documented in previous research (28, 29). Interestingly, there was a greater between-individual variability in RMR in males compared with females, which supports the theory of greater male variability. In contrast, for all other outcomes other than sex hormone concentrations, no evidence for meaningful differences in between-individual or within-individual variance between sexes was observed. Although there was a trend for females to have higher within-individual CV, the SD showed no sex differences, suggesting that any sex difference in within-individual CV is likely not biologically significant.

In addition to potential sex differences in intraindividual variance, there is also the greater male variability hypothesis that proposes that across species and traits, males tend to display greater between-individual variability than females (1, 2). Within our data, we only observed evidence in clear support of this hypothesis for resting metabolic rate. The primary factor that determines resting metabolic rate is fat-free mass (or more specifically body cell mass), with other contributions from fat mass and recent energy balance status (30). Along with greater interindividual variability in physical activity (2), males have been shown to have greater interindividual variability in fat-free mass (2), which may partly explain the greater variability in resting metabolic rate observed in the present analysis. In addition, a positive or negative energy balance in the preceding days can affect RMR and it is possible that gendered behavior around physical activity and eating could play a role (30, 31).

The within- and between-participant standardization of menstrual cycle phase is sometimes used with the aim of minimizing variance in sex hormone concentrations and any subsequent impact on outcomes. It is therefore plausible that the standardization (or not) of menstrual cycle status could influence whether a study demonstrates evidence for greater within-individual variance in metabolic outcomes or not. Two of the studies included in this analysis had no standardization of the menstrual cycle with a washout period of 2–7 days (16; J. J. D. Watkins, H. A. Smith, A. Hengist, S. B. Nielse, U. R. Mikkelsen, J. Saunders, F. Koumanov, J.A. Betts, J. T. Gonzalez, unpublished observations). Although a small sample size, there was no evidence for meaningful increases in within-individual variability of females versus males in these two studies. In fact, the mean and median variability estimates were numerically lower in females compared with males for insulin (16). However, the direction of the effect could change in a larger sample with no menstrual cycle standardization.

Many studies often allow 28 days between trial days for female participants to ensure intraindividual standardization of the menstrual cycle and to standardize endogenous hormone concentrations across trial days. Interestingly, when menstrual cycle phase is controlled within participants, there is still substantially higher within-individual variation in 17β-estradiol and progesterone in females compared with males (18). Moreover, many females do not have a 28-day cycle, with cycle length often varying greatly between cycles (32). Therefore, standardizing the menstrual cycle phase with a 28-day washout period may not ensure individuals have similar 17β-estradiol and progesterone concentrations or that they are tested within the same menstrual phase. These data therefore suggest that within-participant standardization of menstrual cycle phase by self-report is insufficient to eliminate the sex-based within-individual variance in sex hormones within the spread of concentrations in this analysis. Nonetheless, any potential impact on the variability of fasting metabolite and insulin concentrations, resting metabolic rate, and body mass appears negligible for this analysis. Accordingly, it appears that allowing 28 days between repeat testing is sufficient to eliminate potential within-individual variation from the menstrual cycle in the outcomes reported here. However, we do not have data of the phase of menstrual cycle that females were studied in and therefore our data may not capture variability across the whole cycle. It is possible that greater variability may be observed with these standardization techniques if females were studied in different phases of the cycle. In addition, the studies included in this analysis likely involved some postmenopausal females, which may contribute to the lack of sex differences in within-individual variability seen. However, the average age of female participants included in this analysis is 30 yr, suggesting the majority of participants were likely not postmenopausal.

The within-participant, pretesting control of lifestyle (e.g., diet and physical activity) is also a commonly used approach to reduce within-individual variance in metabolic outcome measures. For example, resting metabolic rate can be augmented for up to 14 h following a bout of moderate- to high-intensity exercise (33). In addition, acute periods of energy deficit or low carbohydrate availability can alter glycogen storage and hormonal responses (34). In this analysis, all studies standardized diet for at least 24 h before each trial and all but one study asked participants to refrain from vigorous exercise during the 24 h prior. In this study, the pretrial standardization of diet and exercise that we included may be adequate to eliminate meaningful differences in the within-individual variability of fasting metabolite concentrations between females and males. Researchers using similar pretrial standardization may expect similar within-individual variability but different pretrial standardization may result in higher within-individual variability.

Compared with endogenous sex hormones, hormonal contraception produces more stable concentrations of sex hormones over the period of use (35). If hormonal contraception is used over a study period, it may result in lesser variation in estradiol and progesterone concentrations. The prevalence of hormonal contraceptives in the United Kingdom is ∼26% of females (36) and therefore it is likely that a similar proportion of the population in the studies included here were on hormonal contraception. Only one study characterized the hormonal status of the females (18), and therefore any differences in within-individual variability of metabolic outcomes between hormonal contraceptive use and eumenorrheic females cannot be determined by this present study.

The present study suggests that females do not have greater within-individual variability compared with males. However, this may be due to sampling and the type of participants included in this analysis. The population of females and males recruited for these studies may not be similar with different types of females and males volunteering for the studies. If a broad cross section of males volunteered for the studies and a narrower, more homogenous sample of females volunteered, this may result in a larger or smaller within-individual variability, respectively. In the majority of studies in this analysis, menstrual cycle phase of repeat testing was controlled for which may have contributed to a more homogenous sample of females. Moreover, in females, cyclical changes in plasma TG concentrations have been shown to be greater in individuals with a high physical activity level (37), suggesting within-individual variability may differ depending on participant characteristics. A more homogenous sample may also result from a small sample size not allowing for a broad spectrum of participants. In addition, in this analysis it is possible that individuals participated in more than one study, which could reduce within-individual variability. However, some studies in this analysis had larger sample sizes and likely had a greater spectrum of participants, but overall within-individual variability between sexes remained not significantly different.

### Conclusions

In summary, the present study demonstrates that males can display larger between-individual variance in resting metabolic rate, but does not provide evidence that females show a greater degree of within-individual variation than males in many fasting metabolic outcomes, resting metabolic rate, or body mass. These data were observed in the context of when participants are instructed to standardize diet and physical activity for at least 24 h prior and is despite females showing a higher between-individual and within-individual variability in 17β-estradiol and progesterone concentrations. The data in this present study therefore suggest that—within the context of the studies described—there is no evidence to exclude females on the basis of introducing greater within-individual variance in the variables reported but repeated testing should occur 28 days apart to provide some menstrual cycle phase control. This has profound implications for the interpretation of data from past studies where sex differences were of interest and, moreover, for the design of future studies, where researchers might be unnecessarily focused on one sex rather than the other on the basis of a perceived sex difference in variability.

## DATA AVAILABILITY

Data are available to bona fide researchers upon request at https://doi.org/10.15125/BATH-01393.

## SUPPLEMENTAL MATERIAL

10.6084/m9.figshare.25417468 Supplemental Fig. S1: https://doi.org/10.6084/m9.figshare.25417468.

## GRANTS

This project was funded by the British Heart Foundation (PG/19/43/34432), Arla Foods Ingredients, Lucozade Ribena Suntory, Rank Prize Funds, and Cosun Nutrition Center.

## DISCLOSURES

J. T. G. has received research funding from BBSRC, MRC, British Heart Foundation, Clasado Biosciences, Lucozade Ribena Suntory, ARLA Foods Ingredients, Cosun Nutrition Center, and the Fruit Juice Science Centre; is a scientific advisory board member to ZOE; and has completed paid consultancy for 6d Sports Nutrition, The Dairy Council, PepsiCo, Violicom Medical, Tour Racing Ltd., and SVGC. J. A. B. is an investigator on research grants funded by BBSRC, MRC, British Heart Foundation, Rare Disease Foundation, EU Hydration Institute, GlaxoSmithKline, Nestlé, Lucozade Ribena Suntory, ARLA foods, Cosun Nutrition Center, American Academy of Sleep Medicine Foundation and Salus Optima (L3M Technologies Ltd); has completed paid consultancy for PepsiCo, Kellogg’s, SVGC, and Salus Optima (L3M Technologies Ltd); is Company Director of Metabolic Solutions Ltd; receives an annual honorarium as a member of the academic advisory board for the International Olympic Committee Diploma in Sports Nutrition; and receives an annual stipend as Editor-in Chief of *International Journal of Sport Nutrition & Exercise Metabolism*. None of the other authors has any conflicts of interest, financial or otherwise, to disclose.

## AUTHOR CONTRIBUTIONS

L.B., J.B., and J.T.G. conceived and designed research; L.B. and J.B. analyzed data; L.B. and J.B. interpreted results of experiments; L.B. and J.B. prepared figures; L.B., J.B., and J.T.G. drafted manuscript; L.B., J.B., J.A.B., and J.T.G. edited and revised manuscript; L.B., J.B., J.A.B., and J.T.G. approved final version of manuscript.
